# A Vanishing Polyp: A Large Ileal Lipoma Causing Ileocolic Intussusception

**DOI:** 10.7759/cureus.85833

**Published:** 2025-06-12

**Authors:** Chiaw Yuan Tan, Kosasih Sumitro, Amy Thien, Kian Chai Lim, Aye Aye Tun, Vui H Chong

**Affiliations:** 1 Department of Internal Medicine, Raja Isteri Pengiran Anak Saleha Hospital, Bandar Seri Begawan, BRN; 2 Department of General Surgery, Raja Isteri Pengiran Anak Saleha Hospital, Bandar Seri Begawan, BRN; 3 Department of Radiology, Raja Isteri Pengiran Anak Saleha Hospital, Bandar Seri Begawan, BRN

**Keywords:** colonoscopy, ileal lipoma, intussusception, iron deficiency anemia, polyp

## Abstract

Intussusception is defined as invagination of a proximal segment of gastrointestinal tract into distal segment and is an uncommon pathology. It is more common in children. In the adult population, it is often due to a polyp or tumor. We report an interesting case of a vanishing colonic polyp in a 55-year-old man who presented with a three-month history of intermittent right abdominal pain, altered bowel habit, and weight loss. Colonoscopy demonstrated a large, elongated polyp in the ascending colon, which vanished during attempts to intubate proximally. A computed tomography scan showed a lipomatous polyp in the distal ileum confirmed on a repeat colonoscopy. The patient proceeded with surgical resection, and histology confirmed the polyp to be an ileal submucosal lipoma. There is literature on ileocolic intussusceptions secondary to ileal submucosal lipoma; however, our case is interesting and unique in that the intussusception was seen and reduced during the colonoscopy.

## Introduction

Intussusception is generally uncommon and is reported more in the pediatric population. In the adult population, intussusception accounts for up to 1% of intestinal obstruction, and carcinoma and polyps are identified during perioperative as the lead point of intussusception in at least 90% of cases [1]. Small bowel lipomas, which are typically benign, are mostly located at the distal ileum and are submucosal mesenchymal tumors. They are usually asymptomatic, but lesions that are ≥ 2 cm may cause nonspecific symptoms, such as abdominal pain, diarrhea, bleeding, and obstruction [2]. We report an interesting case of a vanishing elongated polyp in the proximal colon encountered during colonoscopy, which was later found to be due to an ileal lipoma that intussuscepted. He was successfully treated with surgical resection.

## Case presentation

A 55-year-old man presented without any significant past medical history with intermittent right abdominal pain that radiates to the right flank, altered bowel habit, and weight loss of up to 10 kg over a month's duration. This was interposed with a period of no symptoms until they reoccurred suddenly. He denies experiencing any upper gastrointestinal (GI) symptoms, abdominal distention, fever, or any bleeding, altered blood, or fresh bleeding. There was no family history of GI malignancy, and he was a non-smoker. Abdominal examination revealed mild mid-abdominal tenderness but without any mass. Per rectal examination showed no mass and normal stool. Initial laboratory investigation revealed iron deficiency anemia with a level of 8.1 gr/dL (normal > 13 gr/dL), ferritin 10 ng/mL (normal: 30-400 ng/mL); otherwise, renal and liver function and inflammatory markers were unremarkable.

**Figure 1:**
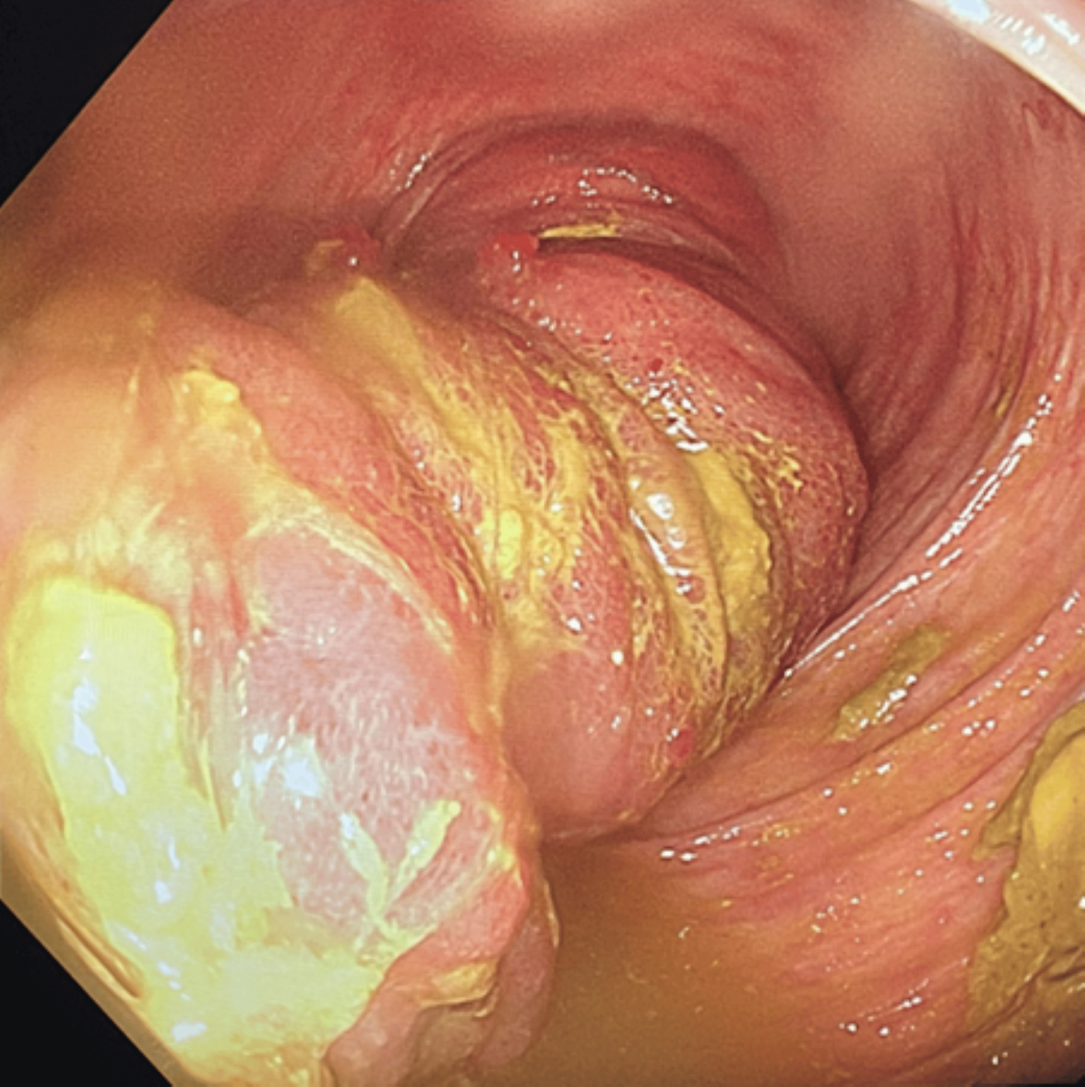
A colonoscopy view in the ascending colon showing a large, elongated polyp.

In view of the symptoms and anemia, GI neoplasm was suspected. He underwent bidirectional endoscopies. Upper GI endoscopy showed only gastritis, and testing was negative for *Helicobacter pylori* infection. Colonoscopy interestingly showed a large, elongated polyp in the proximal colon (Figure 1) on initial intubation. It was not possible to visualize the ileocecal valve and the cecum. Interestingly, while inspecting the ascending colon and during withdrawal of the endoscope, the polyp became smaller and suddenly vanished.

**Figure 2:**
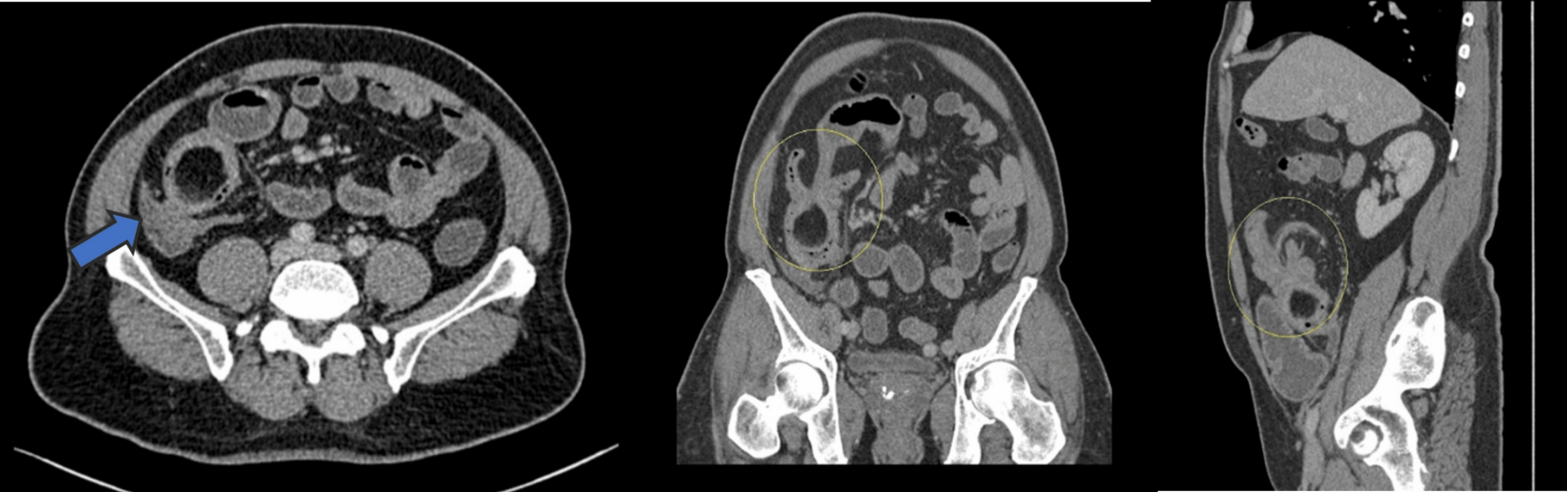
Axial (left), coronal oblique (middle), and sagittal (right) contrast-enhanced CT images showing a round fat-density mass in the distal ileum, with invagination of the ileum and resultant proximal small bowel distension, due to intussusception of ileal lipoma.

The patient remained stable and proceeded with a computed tomography (CT) scan of the abdomen and pelvis. This showed a fat density round mass in the ileum and invagination of the ileal loops in the distal ileum, proximal-to-ileocecal valve, with mild proximal small bowel dilation (Figure 2).

**Figure 3:**
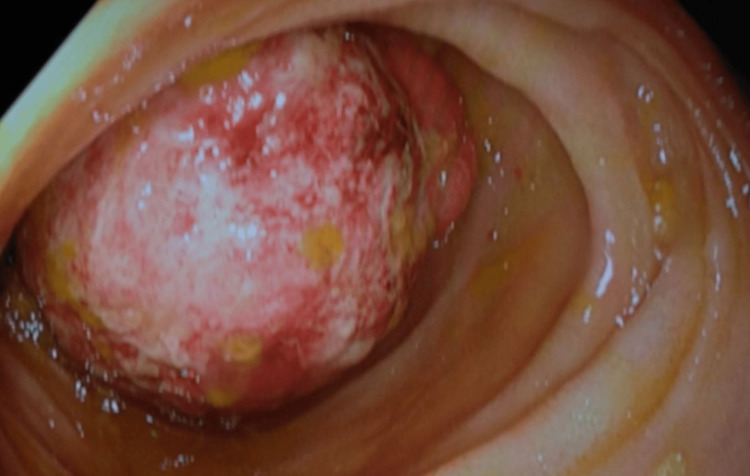
A repeat colonoscopy with ileoscopy showing the polyp with a friable mucosa.

The patient was referred to the surgical department; as his symptoms settled with the spontaneous resolution of the intussusception, the patient was discharged for follow-up. A repeat colonoscopy done two weeks later showed a patulous ileocecal valve, and assessment of the terminal ileum revealed a large polypoidal lesion with friable mucosal surface located at ~15 cm from the ileocecal valve (Figure 3).

**Figure 4:**
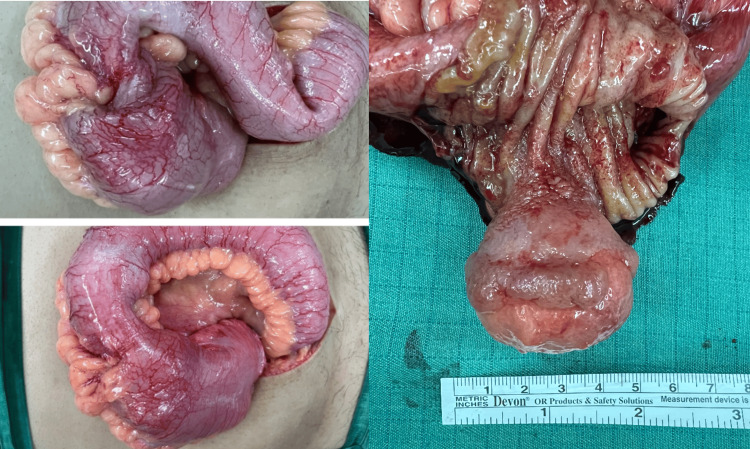
Intraoperative and post-resection images of the pathology.

The patient subsequently underwent surgical resection with primary anastomosis. Intraoperative findings confirmed a 5 x 4 cm mass, located 15 cm from the ileocecal junction (Figure 4). Histopathology findings confirmed the mass to be a lipoma.

He recovered well post-operatively without further bowel symptoms, and his hemoglobin level normalized after four months post-surgery.

## Discussion

We report an interesting case of a vanishing polyp in the ascending colon due to ileo-colic intussusception with a large ileal submucosal lipoma as the leading point. The initial polyp encountered was unusual as it was long and tubular with a very thick stalk. Interestingly, with attempts to get beyond the polyp, the intussusception was inadvertently reduced - the polyp had vanished. Air insufflation and scope manipulations led to the reduction of the intussusceptions, principles used for the management of intussusception - contrast reduction. It was only after the polyp had vanished with a resultant patulous ileocecal valve that intussusception was suspected. There are numerous reports of ileocolic intussusceptions secondary to ileal submucosal lipoma reported in the literature [3,4]. However, our case is interesting in that the intussusception was seen and reduced during the colonoscopy. A lesson from this case is to consider an intussusception when a long tubular polyp is encountered in colonoscopy.

Intussusceptions can be considered uncommon and can be classified according to their location: entero-enteric, ileo-cecal, ileo-colic, and colo-colic, with ileo-colic intussusception being the most common. The most common cause of intussusception in adults is a malignant lesion (65%-75%), followed by a benign lesion such as lipoma, polyp, or lymphangioma (15-20%) [5].

GI lipomas are the second most common type of benign GI neoplasm, followed by adenomas. It is most frequently found between 50 and 70 years old, with an incidence ranging between 0.15% and 4.4%, more commonly in the colon. A vast majority (90-95%) are sub-mucosal, only a small percentage sub-serosal, and can be sessile or pedunculated [2,6]. Colonic lipoma mainly right-sided colon is the most common location, followed by the small intestine (mainly ileal), stomach, and oesophagus [1,7].

Most lipomas are asymptomatic and are found incidentally during endoscopic examination. Typically, these lesions are small. The larger the lipoma, the more likely it is to cause symptoms such as nonspecific abdominal pain, colic, and bowel obstruction, which can be intermittent due to obstruction or intussusception. Large lipomas are more likely to cause intussusception as they can form a leading point. There has also been a case reported for colonic lipoma that had caused intussusception and prolapse out of the rectum.

GI submucosal lipomas larger than 2 cm may develop mucosal ulceration and can present with iron deficiency anaemia [7,8]. As in our case, the patient presented with non-specific abdominal pain with iron deficiency anemia, and no overt GI bleeding. With these symptoms, GI lipoma is diagnosed by barium enema, abdominal CT, or colonoscopy.

Lipoma is managed according to size and symptoms. Small submucosal lipomas not big enough to cause lumen compromise and symptoms are typically left behind and do not require surveillance. Large lipomas can be resected endoscopically, depending on the side and presence of a stalk. However, lipomas larger than 5 cm, as in our case, may act as a leading point for intussusception, causing subacute small bowel obstruction, and surgical intervention is often required. For cases presenting with intussusception, management can be non-surgical at the initial presentation. More commonly reported in the paediatric population, barium enema or air insufflation is often first-line management of intussusception [9,10]. For those with polyps or tumors as leading points, surgery is often required.

## Conclusions

Although lipomas are mostly asymptomatic, but, if large, they may act as a leading point for intussusception in the GI tract; ulceration may also occur, leading to iron deficiency anemia. In cases where a small bowel lipoma becomes symptomatic, resection is the preferred treatment.
